# Sequential neoadjuvant chemoradiotherapy (CRT) followed by curative surgery vs. primary surgery alone for resectable, non-metastasized pancreatic adenocarcinoma: NEOPA- a randomized multicenter phase III study (NCT01900327, DRKS00003893, ISRCTN82191749)

**DOI:** 10.1186/1471-2407-14-411

**Published:** 2014-06-07

**Authors:** Michael Tachezy, Florian Gebauer, Cordula Petersen, Dirk Arnold, Martin Trepel, Karl Wegscheider, Phillipe Schafhausen, Maximilian Bockhorn, Jakob Robert Izbicki, Emre Yekebas

**Affiliations:** 1 Department of General, Visceral and Thoracic Surgery, University Medical Center Hamburg Eppendorf, Martinistr. 52, Hamburg 20246, Germany; 2 Department of Radiotherapy and Radio-Oncology, Hamburg, Germany; 3 University Cancer Center Hamburg (UCCH) - Hubertus Wald Tumor Center, Hamburg, Germany; 4 Department of Medical Biometry and Epidemiology, University Medical Center Hamburg Eppendorf, Hamburg, Germany; 5 Clinic for Medical Oncology, Tumor Biology Center- Freiburg im Breisgau, Breisgau, Germany; 6 Departement of General, Visceral and Thoracic Surgery, Darmstadt Clinic, Darmstadt, Germany

**Keywords:** Pancreas cancer, Surgery, Neoadjuvant chemoradiation, EBRT, Gemcitabine, Randomized trial

## Abstract

**Background:**

Median OS after surgery in curative intent for non-metastasized pancreas cancer ranges under study conditions from 17.9 months to 23.6 months. Tumor recurrence occurs locally, at distant sites (liver, peritoneum, lungs), or both. Observational and autopsy series report local recurrence rates of up to 87% even after potentially “curative” R0 resection. To achieve better local control, neoadjuvant CRT has been suggested for preoperative tumour downsizing, to elevate the likelihood of curative, margin-negative R0 resection and to increase the OS rate. However, controlled, randomized trials addressing the impact of neoadjuvant CRT survival do not exist.

**Methods/Design:**

The underlying hypothesis of this randomized, two-armed, open-label, multicenter, phase III trial is that neoadjuvant CRT increases the three-year overall survival by 12% compared to patients undergoing upfront surgery for resectable pancreatic cancer. A rigorous, standardized technique of histopathologically handling Whipple specimens will be applied at all participating centers. Overall, 410 patients (n = 205 in each study arm) will be enrolled in the trial, taking into regard an expected drop out rate of 7% and allocated either to receive neoadjuvant CRT prior to surgery or to undergo surgery alone. Circumferential resection margin status, i.e. R0 and R1 rates, respectively, surgical resectability rate, local and distant disease-free and global survival, and first site of tumor recurrence constitute further essential endpoints of the trial.

**Discussion:**

For the first time, the NEOPA study investigates the impact of neoadjuvant CRT on survival of resectable pancreas head cancer in a prospectively randomized manner. The results of the study have the potential to change substantially the treatment regimen of pancreas cancer.

**Trial registration:**

Clinical Trial gov: NCT01900327, DRKS00003893, ISRCTN82191749

## Background

Due to anatomical reasons, surgery for pancreatic cancer frequently fails to achieve tumor-free resection margins as the most important prerequisite for prevention from tumor recurrence. This results in insufficient local control that represents, beside early metastatic dissemination, the reason for the catastrophic overall prognosis of the disease. In the GITSG, EORTC, and ESPAC-1 trials, local tumor recurrence was identified the first site of failure in 39%, 53%, and 62% of enrolled patients, respectively [1-3]. Even higher local recurrence rates of up to 87% after “curative” surgery have been reported in observational and autopsy series [4,5]. Recent studies showing that the assessment of R1 status increases substantially to 65%-85% when applying rigorous, fully standardized techniques of handling Whipple specimens [6-8] provide strong evidence that this high incidence of local relapse is due to a tremendous rate of unrecognized, non-curative resections with microscopic involvement of the circumferential resection margin (R1 status). Therefore, the major impetus of neoadjuvant CRT for pancreatic cancer is to increase the rate of curative margin-negative R0 resections, hereby resulting in improved survival.

## Methods/Design

**Figure 1:**
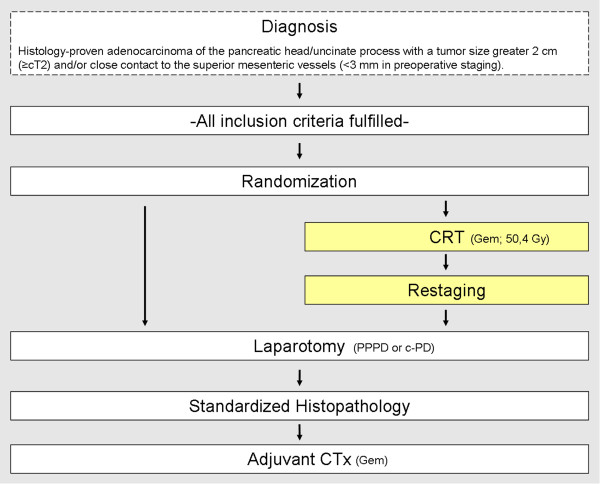
**Trial flow.** Patients are allocated either to intervention or control group after randomization. Patients in the intervention group undergo sequential neoadjuvant CRT (yellow bars) followed by curative surgery. Patients in the control group are subjected to upfront surgery. After completion of the study, subset analysis is performed stratifying patients with resectable vs. borderline resectable lesions. Both groups will receive adjuvant chemotherapy. No postoperative, adjuvant EBRT in patients undergoing upfront surgery (group 2) is performed.

The presented study design is an open labeled randomized controlled phase III trial designed and organized by the Department of General, Visceral and Thoracic Surgery at the University Hospital Hamburg-Eppendorf, approved by the Ethical Review Board of the Medical Association of Hamburg (Ethik-Kommission der Ärztekammer Hamburg) and funded by the Federal Ministry of Education and Research of the Federal Republic of Germany (Bundesministerium für Bildung und Forschung der Bundesrepublik Deutschland).Patients fulfilling eligibility criteria are randomized to either receive neoadjuvant radiochemotherapy followed by surgical resection and facultative adjuvant therapy or receive upfront surgery and adjuvant therapy (Figure 1).

### Inclusion/exlusion criteria

Key inclusion criterion is the biopsy-proven, non-metastasized, adenocarcinoma of the pancreatic head/uncinate process larger than two centimeter in size (≥cT2) and/or in close contact with the mesenterico-portal axis and superior mesenteric artery (SMA) (less than 3 mm). Further inclusion and exclusion criteria are presented in Table 1.

**Table 1:** Inclusion and exclusion criteria

**Key inclusion criteria**	**Key exclusion criteria**
• Histology-proven, resectable adenocarcinoma of the pancreatic head/uncinate process with a tumor size greater 2 cm (≥cT2) and/or close contact to the superior mesenteric vessels (≤3 mm in preoperative staging).	• Age ≤ 18 years
	• Recurrent disease
	• HInfiltration of extrapancreatic organs (except duodenum and transverse colon)
• HNo evidence of metastasis to distant organs (liver, peritoneum, lung, others).	• Persistent cholestasis/cholangitis despite adequate biliary stenting
• Serum creatinine level ≤ 3.0 mg/dl	• HGastric outlet obstruction, especially in the event of endoscopically evidenced tumor invasion into the gastroduodenal mucosa.
• HSerum total bilirubin level ≤ 3.0 mg/dl in the absence of biliary obstruction (In the event of biliary obstruction, patients allocated to the CRT group must undergo interventional endoscopy or percutaneous drainage for biliary decompression. Post-interventionally, bilirubin levels should be ≤ 3.0 mg/dl before patients are subjected to CRT. In control patients undergoing upfront surgery, serum total bilirubin levels ≤ 10.0 mg/dl are tolerated, unless clinical and laboratory signs of severe cholangitis take place. Patients with serum total bilirubin level > 10.0 mg/dl undergo preoperative biliary decompression, preferentially by interventional endoscopy)	• Tumor specific pre-treatment
	• History of gastrointestinal perforation, e.g. perforated colonic diverticulitis, abdominal abscess or intestinal fistula within 6 months prior to potential study participation
	• Radiographic evidence of severe portal hypertension/cavernomatous transformation that may, at the discretion of the participating investigators, hamper surgery
	• Other concurrent malignancies except for basal cell cancer of the skin and in-situ cervical cancer, Premalignant hematologic disorders, e.g. myelodysplastic syndrome
• White blood cell count ≥ 3.5 × 10^9^/ml, platelet count ≥ 100 × 10^9^/ml	• Severe organ dysfunctions (e.g. Liver cirrhosis ≥ Child B; Cardio-pulmonal diseases (NYHA ≥ III, arrhythmia Lown III/IV, global respiratory insufficiency); Ascites; Acute pancreatitis; bleeding diathesis, coagulopathy, need for full-dose anticoagulation or INR > 1.5; other severe diseases that might prevent completion of the treatment regimen)
• HAbility to understand and willingness to consent to formal requirements for study participation	
Written informed consent	
	• Chronic infectious diseases, especially immune deficiency syndromes, e.g. HIV infection, active tuberculosis within 12 months prior to potential study participation
	• History of severe neurologic disorders, e.g. cerebrovascular ischemia
	• History of prior deep venous thrombosis or pulmonary embolism
	• Pregnant or nursing women are ineligible and patients of reproductive potential must agree to use an effective contraceptive method during participation in this trial and for 6 months following the trial
	• Serious medical, psychological, familial, sociological or geographical conditions or circumstances potentially hampering compliance with the study protocol and follow-up
	Participation in other clinical trials during the last 6 months before allocation to trial

### Staging

Baseline diagnostic staging consists of abdominal multi-detector computed tomography (MDCT) according to a standardized protocol. For determination of resectability, MDCT with at least 16 rows applying both oral and intravenous contrast media is performed. MDCT-based imaging focuses on the upper abdomen with native, arterial, and parenchyma phase, where the parenchyma phase should include the pelvis. Imaging criteria derived from the recent consensus definition of the Society of Surgical Oncology, the American Society of Clinical Oncology and the American Hepato-Pancreatico-Biliary Association [9] are applied for preoperative assessment of local resectability.

• Potential Resectability: visualizable fat plane around celiac and superior mesenteric arteries, and patent superior mesenteric/portal vein (SMV/PV).

• Borderline Resectability: substantial superior mesenteric/portal vein impingement, tumor abutment on the SMA < 180°, GDA encasement up to the origin of the hepatic artery, or colonic/mesenteric root invasion (Prior to treatment initiation, patients will be coded as resectable or borderline resectable.)

Randomization-based allocation to one of both treatment arms is performed independently from this stratification. However, subgroup analysis regarding the potential benefits of neoadjuvant CRT will be performed after completion of the trial.

Radiographic workup is completed by chest X-ray. Baseline laboratory and operative eligibility tests (including CA19-9 and CEA assessment) will be performed for every patient in routine preoperative work-up.

Histological proof of adenocarcinoma is mandatory for study inclusion. Therefore, in the event of a suspicious mass in the pancreatic head without evidence of distant metastasis, the eligibility of patients is assessed by endosonography-guided fine-needle aspiration (EUS-FNA). Explorative laparoscopy/laparotomy may alternatively be performed when EUS-guided biopsy is technically not feasible, e.g. due to previous gastric surgery with diversion of the gastroduodenal continuity, or when histologic findings are inconclusive.

Patients are considered eligible and checked for fulfilment of inclusion and exclusion criteria. Patients, who meet inclusion criteria, are asked for study participation after appraisal of all findings in a multidisciplinary tumor board. Randomization is performed after patients have signed written informed consent.

### Radiochemotherapy

Patients allocated to undergo neoadjuvant CRT will receive Gemcitabine 300 mg/m^2^/weekly for 6 weeks combined with 3-D-conformal external beam radiotherapy (EBRT) according to the following protocol:

Clinical Target Volume (CTV) is defined based on standardized, preoperative MDCT imaging with frontal and sagittal reconstruction of CT scans. For better differentiation of the small bowel, celiac trunk vessels, superior mesenteric artery, mesenterico-portal axis, and neighboring organs, oral contrast ingestion and intravenous contrast enhancement is performed. Conventionally fractionated irradiation with a total dose of 50.4 Gy is delivered over 28 days in 1.8 Gy fractions targeting also frequently involved, peripancreatic “at risk” lymph nodes (Group 8, 10, 11, 13, 14, 16, 17) including an additional safety margin of 1.5 cm. EBRT is performed from Monday to Friday resulting in a total treatment time of 38 days if protocol starts on Monday. Dose limits for organs at risk are ≤ 30 Gy to 50% of the liver volume, ≤ 20 Gy to 50% of the right kidney volume, ≤ 20 Gy to 30% of the left kidney volume, and ≤ 45 Gy to the spinal cord.

Depending on the clinical condition, surgery is scheduled within a time frame of 3 (earliest) to 6 weeks (latest) after completion of neoadjuvant CRT.

### Surgery

Patients allocated to the reference group undergo primary surgery in curative intent. Both study arms undergo, depending on the individual tumor site, either pylorus-preserving pancreatoduodenectomy (PPPD) or classic partial pancreatoduodenectomy (cPD) with standard lymphadenectomy (SLA). Intraoperative frozen-sections will be routinely performed from the pancreatic transection surface and the common bile duct (CBD). SLA includes lymph node dissection at the anterior and posterior aspect of the pancreatic head, the pylorus, the hepatoduodenal ligament, the right aspect of the SMA, and the hepatic artery in its course at the superior part of the pancreatic head. For surgical quality control, post-resectional photo-documentation is requested.

In cases of unexpected intraoperative unresectability (distant metastasis, tumor invasion into the celiac trunk, SMA, mesenteric root), patients undergo suitable bypass procedures. This is followed by palliative CTx, e.g. Gemcitabine 1000 mg/m^2^ 6 cycles at day 1, 8, 15 of each 28-day cycle plus/minus Erlotinib.

Irrespective of whether or not neoadjuvant CRT has been performed, all patients undergo adjuvant CTx, preferentially with Gemcitabine 1000 mg/m^2^ 6 cycles at day 1, 8, 15 of each 28-day cycle.

### Histopathological examination

To minimize inter-institutional bias in histopathologic reporting, standardized handling of Whipple specimens is essential. In line with recent developments, guidance will be given regarding the extent and technique of tissue sampling, and microscopic involvement will be defined by a minimum clearance of 2 mm [10]. To ensure accurate histopathology reporting including margin status assessment, the participating study centres will follow a standardized protocol with axial slicing of the Whipple specimen and extensive tissue sampling at all circumferential margins. Histopathology results, especially regarding the R-status, may be re-evaluated at the discretion of two experts in pancreatic pathology in any participating center in a blinded fashion at regular intervals [6,7]. This designated panel of histopathologists will ensure that (1) the agreed pathology protocol meets the set standards in terms of accuracy and reproducibility and (2) that the protocol is uniformly applied in all centers. The pathology protocol will in particular address the specific diagnostic challenges that are posed by tumour regression following pre-operative CRT.

### Follow up

**Figure 2:**
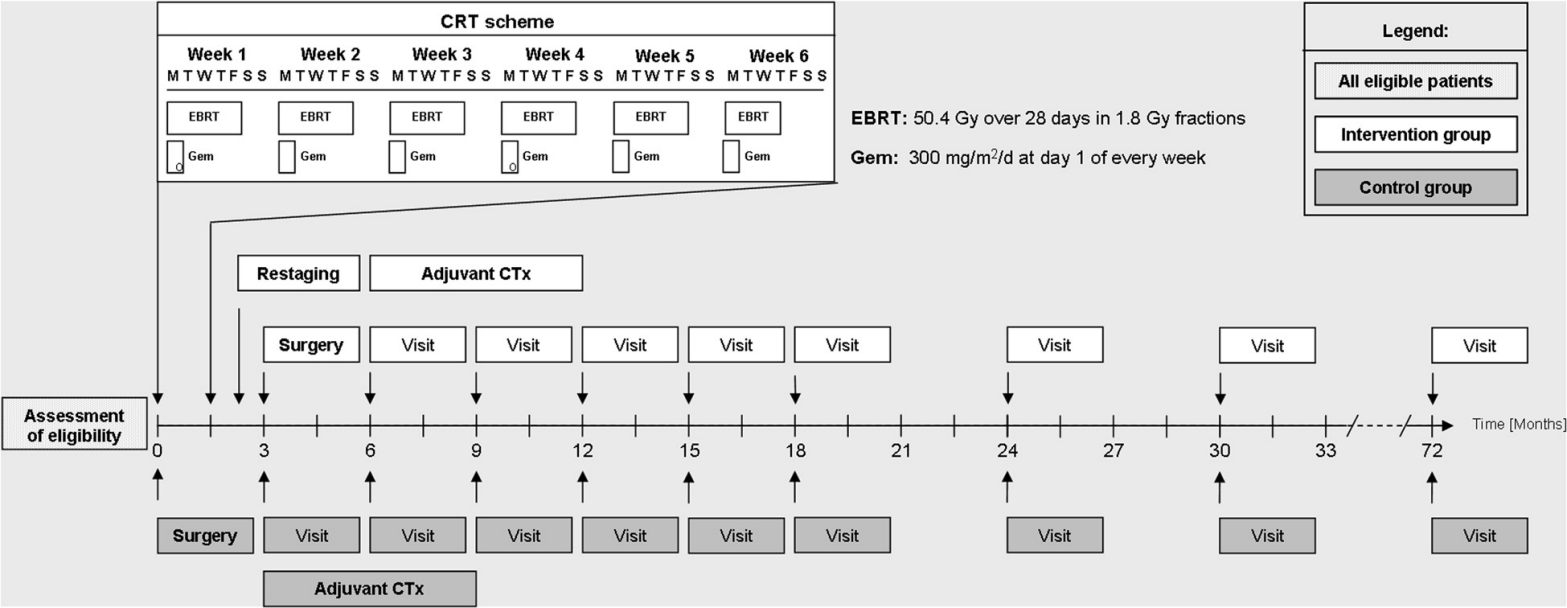
**Follow-up and study visits.** Study visits will be performed in 3-months intervals within the first 18 months and in 6-months intervals thereafter. Total follow-up will be 24 months after end-of-recruitment or 36–72 months after randomization.

Study visits with alternating ultrasound and MDCT investigations are performed in 3 month intervals for 18 months post-operatively, and in 6 month intervals thereafter (Figure 2). Study visits include clinical examination (Karnofsky-index) and routine laboratory investigations including tumor marker assessment (CEA, CA 19–9). Either MDCT or abdominal ultrasound imaging is scheduled according to the following flow:

• MDCT imaging and chest X-ray at 6, 12, 18, 24, 30, 36 months after randomization. Additional MDCT scanning may be performed in the event of suspected tumor recurrence

• Abdominal ultrasound at 3, 9, and 15 months after randomization.

Randomized patients in whom either curative resection was intraoperatively not feasible or in whom follow-up investigations evidences tumor recurrence at any site (local, distant, both) will undergo follow-up study visits. Any palliative therapy and supportive means will be documented and annotated in CRF sheets. In contrast to patients undergoing curative surgery, imaging investigations, especially MDCT, will be conducted only for medical reasons, i.e. routine follow-up MDCT will be omitted.

### Study objective

Efficacy of neoadjuvant CRT in improving 3-year survival probability from 30% in the control arm undergoing upfront surgery without neoadjuvant CRT to 42% (relative increase of 40%) in the study arm undergoing CRT. The underlying guess of a 30% 3-year survival probability in the control group derives from an assumed median overall survival (MOS) of 20.7 months which corresponds with a MOS of 17.9 months to 23.6 months reported in several randomized trials.

### Primary and secondary endpoints

Primary endpoint of the study is the survival time of the patients, precisely the three year overall survival starting from study inclusion/randomization. Key secondary endpoints are the following:

• Histology-proven R0 resection rate based on a standardized histopathological handling of the surgical specimen.

• Frequency of moderate and severe toxicity events and drop-out rate due to therapy related toxicity (NCI Common Toxicity Criteria v2.0)

• Resectability rate (Note: includes both R0 and R1 resection status).

• Rate of unexpected intraoperative irregularities, operative time, blood transfusion requirement, postoperative morbidity rate, especially that of pancreatic fistula, and mortality rate

• Rate of patients with severe postoperative complications (postop. recovery > 8 weeks) rendering adjuvant treatment worthless

• Disease progression during neoadjuvant therapy

• Quality of life analysis (EORTC QLQ C30 questionnaire)

• Median disease-free survival (DFS, local and distant), overall survival (OS)

• First site of tumor recurrence

### Proposed sample size/power calculations

The sample size calculation is based on the assumption that the 3-year survival probability in the control arm is 30% corresponding to a median overall survival (MOS) of 20.7 months, which is in line with MOS of 17.9 months to 23.6 months reported in clinical trials [3,11-13]. The sample size calculation is based on both a scrutinized literature research and own data sampled in a prospective pancreas cancer database (Department of General, Visceral and Thoracic Surgeryat the University of Hamburg). Overall, this database encompasses 1023 patients operated on for pancreatic cancer from 1/1993 to 12/2010, whose survival data are available. Part of these data has been published [14-16].

Reliable data from phase III trials regarding outcome after sequential CRT followed by surgery for pancreas cancer do not exist. Different neoadjuvant regimens, the lack of clear inclusion criteria (pooling of potentially resectable tumours with borderline resectable and unresectable, locally advanced lesions), and disparities in the accuracy of histopathological evaluation may be the cause for the wide range of reported survival [17]. Therefore, reported MOS in these series ranging from 23.0 months to 34.0 months and 3-year survival probability ranging from 0% to 48% have to be interpreted with caution [18-28].

The trial is expected to be able to demonstrate an absolute (relative) increase of 12% (40%) to 42% 3-year survival with a power of 80%. For this purpose, a group-sequential design with 4 stages spending a total alpha of 5% is considered. A sample size calculation was performed using ADDPLAN 5.0. According to this calculation, 398 fully evaluable patients have to be included.

### Quality control

The procedures set out in this study protocol are designed to ensure that the sponsor and the investigator abide by the principles of the ICH Guideline for GCP (E6) and the Declaration of Helsinki concerning the conduct, evaluation and documentation of the study. The study will also be performed adhering to the local legal conditions and regulations and to the applicable regulatory requirements.

To ensure comparability of tumor characteristics in both treatment arms, allocation of patients is performed by 1:1 randomization regarding both the entire trial and the respective centres using a computerized online randomization tool. Trial site effects are not expected because all participating centres are known to have a high case load in pancreatic surgery. The study design does not enable to blind patients before surgery. However, the trial statistician will check the final analysis of the trial results for statistical correctness based on an anonymized database.

A standardized histopathology protocol as a mandatory prerequisite of the trial will be applied to minimize inter-institutional disparities, in particular regarding circumferential resection status (CRM; R0 vs. R1). To avoid bias in the histopathological examination, the respective pathologist is unaware of whether or not CRT has been performed.

All withdrawals will be documented including the reason. All data of patients who withdraw, e.g., who are discontinued prematurely will be documented and discussed, as necessary, in the final report of the trial.

All available data will be included in the analyses and will be summarized as far as possible. Every effort will be undertaken to fulfill all protocol requirements regarding the collection and management of data. Missing data will be dealt with on an individual basis and will be subject to data queries. Unless otherwise specified, there will be no substitution of missing data. Missing data will not be replaced but will be handled as ‘missing’ in the statistical evaluation.

An advisory Data and Safety Monitoring Board (DSMB) evaluates the risk to the patients on the accrued data provided by the sponsor and the monitor. The multicentre character of the study poses special challenges as to quality control performed on an ad hoc or real-time basis or quarterly, as appropriate. The DSMB will especially focus on inter-institutional differences in diagnostic work-up, clinical staging accuracy, surgery, histopathology reporting, and non-surgical therapy. A further major task of the DSMB is to check for potentially elevated surgical morbidity in CRT patients (intraoperative blood loss, anastomotic leakage rates, etc.).

## Discussion

Randomized trials evidencing the superiority of sequential CRT and surgery strategies over surgery alone do not exist. Nonetheless, in several centers predominantly in the U.S. (in contrast to European centers), neoadjuvant CRT followed by surgery has meanwhile become a “standard” approach towards patients with resectable pancreas cancer. Without clear evidence for the superiority of such an attitude compared to surgery alone, different treatment schedules have been evaluated and are actually under investigation (NCT00049348, NCT00089024, NCT0042673, NCT00557492, NCT00456599).

The scientific rationale for neoadjuvant CRT derives from 2 aspects: EBRT for local control and CTx for both, sensitizing EBRT and early treatment of occult distant metastasis. It is more likely to be completed in its full course (80%-90%) when administered preoperatively, whereas between 21% and 35% of patients may not complete adjuvant treatment due to inadequate postoperative recovery or even never receive such a treatment due to severe postoperative morbidity [29]. Recovery from surgery may especially be delayed when pancreatic fistula constituting the most frequent surgical complication after PD arises. A potential “benefit” of preoperative CRT is in this context that the prevalence of pancreatic fistula formation after PD is, as a result of fibrotic alterations of the gland, lower after preoperative radiation [25,30]. The comparison of the rate of patients with severe surgical morbidity precluding adjuvant CTx, which is planned in both treatment arms, is therefore a secondary endpoint of this trial.

Consistently, most trials on pancreatic cancer report that R0 status is, as compared to R1 patients, not an independent, beneficial prognostic factor. Based on recent studies clearly evidencing that histopathology tends to understage potential involvement of circumferential resection margins if specimens are processed by non-standardized techniques, there is growing awareness that most relevant studies on curatively resected pancreas cancer are significantly biased by substantial weaknesses in the histopathological staging of Whipple specimens. This concerns approximately 60%-80% of anticipated R0 patients, who are underreported and would be staged “R1” when applying standardized pathological handling procedures [6]. Application of a standardized histopathologic protocol known to increase staging accuracy [6,8,31] is a crucial methodological part of this application. Based on these considerations, the potential impact of CRT on circumferential margin status represents a substantial secondary endpoint of this application.

In examining published and ongoing series addressing the benefit of neoadjuvant CRT for resectable pancreatic cancer on a whole, it is important to state that no consensus exists regarding radiation and CT regimens. This reflects both the wide range of applied agents (5-FU, Cisplatin, Gemcitabine, Mitomycin C, Bevacizumab, Capecitabine, Docetaxel, Oxaliplatin) and investigated radiation doses (30 Gy to 50.4 Gy) with fairly wide treatment fields including prophylactic regional nodal radiation.

Chemomodulation with 5-FU has shown to increase survival in irradiated *unresectable,* locally advanced pancreatic cancer (LAPC) [32]. In the neoadjuvant setting, the majority of trials evaluated 5-FU- or Gemcitabine-based CRT, combined with or without Cisplatin [23,27,33-37]. Current results of neoadjuvant Gemcitabine-based CRT in borderline *resectable* LAPC show good tolerability, and a meaningful overall survival of 31 to 34 months was reported for patients receiving resection after induction treatment [23,27]. Furthermore, Gemcitabine-based CRT was feasible and well tolerated in the postoperative setting, also in comparison to adjuvant Gemcitabine alone [37]. In the recent trials by Wilkowski and colleagues, further escalation of Gemcitabine based CRT by Cisplatin (compared to 5-FU based CRT) in unresectable LAPC showed significantly higher grade 3–4 RTOG toxicity without improvement of survival rates [33]. Whereas in the adjuvant or palliative setting CRT is mainly up to 50 Gy, reported preoperative protocols range from 30 to 50.4 Gy in 1.8-3 Gy fractions combined with Gemcitabine 100–1000 mg/m^2^ weekly [23,24,26,27,34,37-44]. With respect to these data, a CRT schedule with weekly doses of Gemcitabine 300 mg/m2 and 50.4 Gy in 1.8 Gy fractions was selected.

After curative resection, the CONKO-001 trial showed that Gemcitabine significantly improved disease free survival (DFS) compared to observation alone [13]. Similar results were obtained in a Japanese phase III trial, showing significant increased DFS and a trend towards better overall survival [45]. The RTOG 97–04 compared systemic 5-FU to Gemcitabine for 3 weeks before and 12 weeks after CRT with 5-FU revealing a trend towards better survival in the Gemcitabine arm [46]. Although the ESPAC-3 trial did not identify any survival benefit of Gemcitabine compared to 5-FU, Gemcitabine showed better tolerability and lower incidence of sever adverse events [11]. Therefore, Gemcitabine is regarded as the current standard for adjuvant treatment and will be administered in both treatment arms for adjuvant therapy [47].

Despite the questionable radiosensitivity and chemosensitivity of the disease, there are 2 strong arguments in favour of neoadjuvant CRT. The first is that negative microscopic resection margin status (R0) as the predominant prerequisite for long-term, local control is in the majority of patients (>50%) not achieved by surgery alone. Advocates of CRT argue that this “limitation” of any surgical therapy is addressed by the radiation “component”, although the impact of potentially increased curative R0 rates on outcome is still largely unknown. Secondly, it appears to be conclusive to administer preoperatively systemic CTx for occult distant metastases non visualizable by current diagnostic imaging tools [21,23,48,49]. Despite of these considerations, scientific evidence that neoadjuvant CRT followed by surgery beneficially impacts survival compared to surgery alone has not been provided. Case series report median OS following neoadjuvant therapy from 10 to 36 months [27,50,51]. The fact that despite the lack of controlled, randomized studies, many institutions, especially in the U.S., subject pancreas cancer patients to neoadjuvant CRT underlies the fundamental need for this trial.

## Competing interests

The authors declare that they have no competing interests.

## Authors’ contributions

This study was designed by EY, MiT and FG. The article was written by MiT and FG. KW performed the sample size calculation and planned the statistical analyses. CP, DA, MaT, PS, MB and JRI are involved in trial implementation and critically revised the manuscript. All authors have read and approved the manuscript.

## Pre-publication history

The pre-publication history for this paper can be accessed here:

http://www.biomedcentral.com/1471-2407/14/411/prepub
